# Stanniocalcin-1 promotes tumor angiogenesis through up-regulation of VEGF in gastric cancer cells

**DOI:** 10.1186/1423-0127-18-39

**Published:** 2011-06-14

**Authors:** Ling-fang He, Ting-ting Wang, Qian-ying Gao, Guang-feng Zhao, Ya-hong Huang, Li-ke Yu, Ya-yi Hou

**Affiliations:** 1 Immunology and Reproductive Biology Lab, Medical School & State Key Laboratory of Pharmaceutical Biotechnology, Nanjing University, Nanjing, PR China; 2 First Department of Respiratory Medicine, Nanjing Chest Hospital, 215 Guangzhou Road, Nanjing, PR China

**Keywords:** STC-1, angiogenesis, VEGF, PKCβII, ERK1/2

## Abstract

**Background:**

Stanniocalcin-1(STC-1) is up-regulated in several cancers including gastric cancer. Evidences suggest that STC-1 is associated with carcinogenesis and angiogenic process. However, it is unclear on the exact role for STC-1 in inducing angiogenesis and tumorigeneisis.

**Method:**

BGC/STC cells (high-expression of STC-1) and BGC/shSTC cells (low- expression of STC-1) were constructed to investigate the effect of STC-1 on the xenograft tumor growth and angiogenesis *in vitro *and *in vivo*. ELISA assay was used to detect the expression of vascular endothelial growth factor (VEGF) in the supernatants. Neutralizing antibody was used to inhibit VEGF expression in supernatants. The expression of phosphorylated -PKCβII, phosphorylated -ERK1/2 and phosphorylated -P38 in the BGC treated with STC-1protein was detected by western blot.

**Results:**

STC-1 could promote angiogenesis *in vitro *and *in vivo*, and the angiogenesis was consistent with VEGF expression *in vitro*. Inhibition of VEGF expression in supernatants with neutralizing antibody markedly abolished angiogenesis induced by STC-1 *in vitro*. The process of STC-1-regulated VEGF expression was mediated via PKCβII and ERK1/2.

**Conclusions:**

STC-1 promotes the expression of VEGF depended on the activation of PKCβII and ERK1/2 pathways. VEGF subsequently enhances tumor angiogenesis which in turn promotes the gastric tumor growth.

## Background

Development of gastric cancer involves multiple factor changes that lead to the transformation of human gastric epithelial cells to gastric cancer cells [1]. Angiogenesis is a critical hallmark of malignancy and can occur at different stages of the tumor progression [2]. Acquisition of the angiogenic phenotype can result from genetic changes or local environmental changes such as the secretion of pro-angiogenic growth factors by tumor that lead to the activation of endothelial cells. Stanniocalcin-1(STC-1) is a glycoprotein hormone originally discovered in the corpuscles of Stannius of bony fish [3]. The expression of the mammalian STC-1 was found in numerous developmental and pathophysiological processes [4-8]. Growing evidence suggests that the mammalian STC-1 may be associated with carcinogenesis. Aberrant STC-1 expression has been reported in breast and ovarian cancers [9-11]. Our previous study found that STC-1 gene could be activated in human gastric cancer BGC823 cells with over-expressed midkine [12]. Midkine is a heparin-binding growth factor, which was highly expressed in various malignant tumors and the increased expression of midkine was significantly associated with the advanced clinical stage and distant metastasis of gastric cancer [13].

Recent works indicated that STC-1 may be involved in the control of the angiogenic process [14]. In colon cancers, STC-1 was highly expressed during angiogenesis and the increased expression of STC-1 may be contributed primarily by the tumor vasculature [15]. VEGF is an important angiogenetic factor and stimulates the proliferation and migration of endothelial cells [16]. Many studies have verified that the expression of STC-1 is related with VEGF [17,18]. Moreover, several reports have shown that PKC plays an important role in regulating VEGF expression in angiogenesis process [19,20]. ERK [21-23], STAT3 [24], P38 and JNK [25] signaling pathway are also involved in the positive control of VEGF expression. However, the exact role for STC-1 in inducing both tumorigeneisis and angiogenesis in cancer is not well understood.

In our present study, we found that STC-1 can promoted angiogenesis *in vivo *and *in vitro*. Moreover, we validated that VEGF is a key angiogenesis factor in STC-1 induced angiogenesis. Furthermore, PKCβII and ERK1/2 signaling pathway mediated STC-1-regulated VEGF expression. We conclude that STC-1 can increase VEGF expression to promote angiogenesis depended on PKCβII and ERK1/2 signaling pathway.

## Results

### STC-1 promotes tumor proliferation and angiogenesis *in vivo*

**Figure 1:**
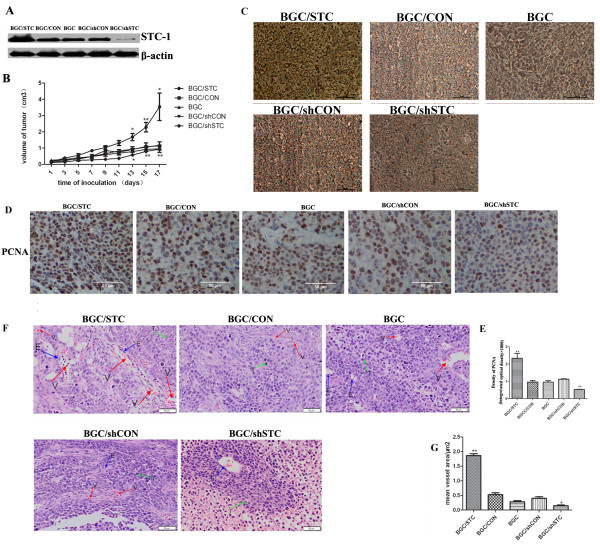
**Tumorigenesis and angiogenesis of BGC cells in nude mice**. (A) Western blotting analysis of the expression of STC-1 in BGC after stable transfection. (B) Mean volumes of the tumor in each group were calculated. Cultured BGC cells and BGC stable transfection cells (10^6 ^cells) were injected subcutaneously into the flank of female nude mice. Tumor volumes were measured and calculated once every two days after we can see the tumor in the flank of nude mice. (C) Immunohistochemical staining of STC-1 in tumor tissues of nude mice. STC-1 was detected on the membrane of tumor cells (D) Immunohistochemical staining of PCNA in tumor tissues of nude mice. PCNA was detected in the nucleus of tumor cells. (E) Quantification of PCNA expression(the Integrated Optical Density (IOD) of PCNA) by image pro-plus software. All histology was carried out on multiple sections from individual mice and three independent in vivo experiments. (F) Hematoxylin and eosin stained sections of the Matrigel plugs (E, endothelial-like cells; T, tumor cells; S, surrounding tissues; V, microvessels). (G) Mean vessel area was quantified in each group. (*P < 0.05, **P < 0.01).

We successfully constructed BGC/STC cell and BGC/shSTC cell line. SiRNA#2, the most effective inhibitor, was used to construct Psilencer4.1™/STC-1 plasmids (Additional file 1 Figure S1A). STC-1 cDNA obtained from gastric tissues were used to construct pcDNA3.1/STC-1 plasmids. STC-1 expressions in BGC823 and transfected BGC823 cells (BGC/CON cell, BGC/STC cell, BGC/shCON cell and BGC/shSTC cell) were confirmed in both mRNA and protein (Additional file 1, Figure S1B, Figure S1A) level. All these cells were cultured under standard culture conditions for 24 h, and found to exhibit the same morphology (Additional file 1, Figure S1C). Afterward, we analyze the tumorigenicity of these stable transfectant in vivo. BGC823 cells and transfected BGC823 cells were injected into the flank of nude mice, and these mice were named as BGC mice, BGC/CON mice, BGC/STC mice, BGC/shCON mice and BGC/shSTC mice. Tumor volumes were measured and calculated. The results showed that the tumor volumes were significantly larger in BGC/STC mice and extremely smaller in BGC/shSTC mice compared with those in BGC mice (Figure 1B). And STC-1 protein expression level was stronger in BGC/STC mice and lower in BGC/shSTC mice (Figure 1C).

We then investigated whether the proliferation of tumor cells was associated with STC-1 expression *in vivo*. The density of PCNA, a proliferation marker of tumor cells, was evidently higher in tumor tissues from BGC/STC mice and lower in tumor tissues from BGC/shSTC mice than that from BGC mice, BGC/CON mice or BGC/shCON mice (Figure 1D, E). However, the proliferation and cell apoptosis of BGC cell, BGC/CON cell, BGC/STC cell, BGC/shCON cell and BGC/shSTC cell had no significant change in *vitro *(Additional file 1, Figure S1D, S1E). The *in vivo *and *in vitro *experiments suggest that STC-1 may promote tumorgenesis through other mechanism, other than tumor cell proliferation itself.

It has been known that angiogenesis have an important role in tumor growth. So we checked the angiogenesis in *vivo*. The results showed that the vascularity was increased in BGC/STC mice and reduced in BGC/shSTC mice compared to BGC mice or BGC/CON mice (Figure 1F, G). This indicated that STC-1 may promote the tumor growth in *vivo *depended on tumor angiogenesis.

### Effects of STC-1 on HUVEC proliferation, migration and tube formation *in vitro*

**Figure 2:**
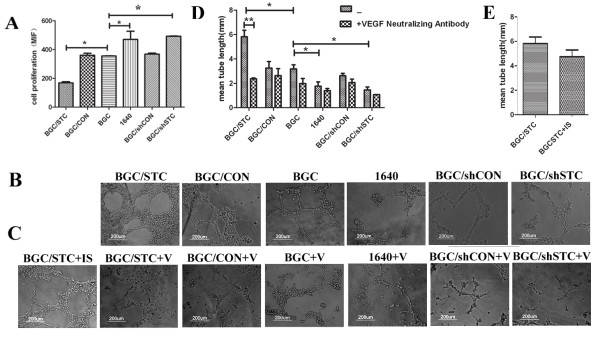
**Effects of STC-1 and VEGF on HUVEC cell proliferation, tube formation**. (A) CFSE positive cells were gated and CFSE fluorescence intensity was showed in histograms. HUVEC were seeded in 12-well plates in triplicate and incubated with different culture supernatants. After 72 h, HUVEC proliferation was detected by FACS. (B) Tube formation of HUVEC induced by different culture supernatants was photographed under a microscope at ×100 magnification. (C) Effects of VEGF on tube formation of HUVEC. Tube formation of HUVECs was photographed under a microscope at ×100 magnification. (D) Mean tube length was quantified by image pro-plus software. All histogram was carried out on multiple sections and the results are representative of three independent experiments. (E) effect of isotype antibody on cell migration. IS: isotype antibody; V:VEGF neutralizing antibody; BGC/STC+IS: BGC/STC cell supernatants added with isotype antibody; BGC/STC+V: BGC/STC cell supernatants added with VEGF neutralizing antibody.

**Figure 3:**
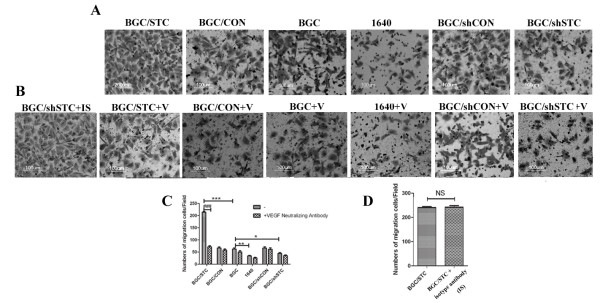
**Effects of STC-1 and VEGF on HUVEC cell migration**. (A) Effects of STC-1 on HUVEC migration. HUVEC were seeded in triplicate on inserts, and incubated for 12 h with different conditioned supernatants. (B) Effects of VEGF on HUVEC migration. HUVEC were seeded in triplicate on inserts, and incubated for 12 h with tumor supernatants incubated with 2 μg/mL VEGF monoclonal antibody (Bioactive). (C) The number of migration cells was quantified under a microscope at ×100 magnification. All histogram was carried out on multiple sections and the results are representative of three independent experiments. (D) Effect of isotype antibody on cell migration. IS: isotype antibody; V:VEGF neutralizing antibody; BGC/STC+IS: BGC/STC cell supernatants added with isotype antibody; BGC/STC+V: BGC/STC cell supernatants added with VEGF neutralizing antibody.

To determine the effect of STC-1 on angiogenesis, we use CFSE staining to detect proliferation rate of HUVECs. We found that BGC/STC culture supernatants could significantly promote HUVEC proliferation, while BGC/shSTC culture supernatants could inhibit HUVEC proliferation (Figure 2A). Then we considered whether the culture supernatants could regulate the migration of HUVEC. The Millicell cell culture insert was used to study the migration of the HUVEC in vitro. The migration of HUVEC was significantly enhanced with BGC/STC medium cultured, while the migration was reduced with BGC/shSTC medium cultured (Figure 2B, D). Tube formation assay was further verified the effect of STC-1 on this angiogenesis process. The formation of tube or cordlike structure could be induced by all kinds of tumor cell supernatants cultured with HUVEC, but not 1640 medium. Notably, BGC/STC supernatants showed an augmentation effect on the tube network while BGC/shSTC supernatants resulted in shorter and more blunted tubes (Figure 3A, C). These results suggest that STC-1 may change some factors of tumor microenvironment to modulate angiogenesis.

### VEGF is neceseary to STC-1 promoting angiogenesis

**Figure 4:**
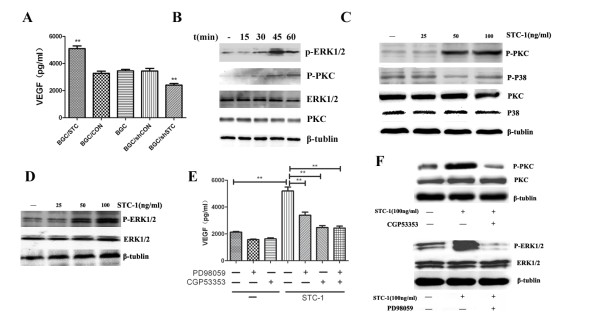
**STC-1 promoted VEGF expressing through PKCβII signaling pathway**. (A) VEGF expression in the different culture supernatants. ELISA assay was used to detect VEGF expression in the culture supernatants. (B) Time courses of PKCβIIand ERK1/2 avtivation induced by STC-1. BGC823 were treated with 50 ng/mL STC-1 for 15, 30, 45, 60 min. Whole- cell lysates were prepared and immunoblotted with antibodies to phosphor-PKC βII, total PKC βII, phosho-ERK1/2 and total ERK1/2. (C) Concentration courses of PKC βIIand P38 activation induced by STC-1. BGC823 were treated with different concentrations STC-1 for 45 min. Whole- cell lysates were prepared and immunoblotted with antibodies to phosphor-PKC βII, total PKC βII, phosho-P38 and total P38. The results are representative of three independent experiments. (D) Concentration courses of ERK1/2 activation induced by STC-1. BGC823 were treated with different concentrations STC-1 for 45 min. Whole- cell lysates were prepared and immunoblotted with antibodies to phosho-ERK1/2 and total ERK1/2. The results are representative of three independent experiments. (E) Effect of STC-1 on VEGF is mediated through PKCβII and ERK1/2 signaling. BGC823 was exposed to either CGP53353 (0.5 μM) or PD98059 (25 μM) for three hours and then individually with STC-1 for 24 h. The results are representative of three independent experiments. VEGF expression in BGC823 cell culture supernatants was determined by ELISA. (F) CGP53353 and PD98059 could inhibit PKC βIIand ERK activation, respectively.

It is well known that VEGF is one of the most common promoters of angiogenesis, as an angiogenetic factor [16], so we investigated whether STC-1 could regulate the expression of VEGF in the gastric cancer cell. We found that ectopic-expression of STC-1 could promote VEGF production in the gastric cancer cell (Figure4A). Moreover, the same result can be obtained when STC-1 protein was added to culture media (Figure4E). However, when VEGF neutralizing antibody was used to neutralize VEGF in the culture supernatants of HUVEC cells, the tube formation (Figure2C, D) and cell migration(Figure3B, C) of the cell induced by STC-1 were markedly abolished in vitro. This means that VEGF indeed promoted the process of angiogenesis. Isotype antibody was used to further confirm that VEGF play an important role in the process of STC-1 regulated angiogenesis (Figure 2E, 3D).

### STC-1 promotes VEGF expression primarily through PKCβIIand ERK1/2 signaling pathway

To understand the regulation of VEGF expression by STC-1, we investigated the main signaling pathways related to VEGF expression. We found that STC-1 could activate both PKCβII and ERK1/2 pathways in time- ang concentration-dependent patterns (Figure4B, C, D). Then we used the PKC and ERK1/2 inhibitor, CGP53353 and PD98059 respectively, to check which pathway related to VEGF expression enhanced by STC-1, and found that VEGF expression can be strongly inhibited by one or both of these inhibitors was used (Figure 4E, F).

## Discussion

Many studies previously have uncovered the biological functions of STC-1 in mammals [3,4]. It is found to be highly expressed in many cancers, such as gastric cancer, colon cancer, ovarian cancer and breast cancer [9-11,26,27]. These observations suggest that STC-1 might play an important role in cancer development. In this study, we for the first time showed that STC-1 enhances the expression of VEGF in gastric cancer cells and promotes tumor growth through enhancing tumor angiogenesis.

The effect of STC-1 on cell proliferation is still controversial. Wu et al. found a direct inhibitory effect of STC-1 on mammalian longitudinal bone growth [28] while Liang et al reported that down-regulation of STC-1 enhanced the proliferation of breast cancer cell lines. However, a recent study showed that over-expression of STC-1 in ovarian cancer cells enhanced cell proliferation, migration, and tube formation *in vitro *and increased the growth of xenograft tumors in mice [29]. In this study, we found that STC-1 had no effect on BGC cell proliferation *in vitro*. However, it significantly promoted tumor growth *in vivo*. This suggests thatSTC1-induced tumorigenesis is not through enhancing cell proliferation directly. There might be other mechanisms that promote tumorigenesis. It is well known that the development of tumors is dependent upon neovascularization [30,31]. Previous studies have proved that STC-1 is highly expressed in tumor vasculature in breast adenocarcinomas and colon cancers [26,32]. A recent study by G. Basini et al. reported that STC-1 might be involved in the angiogenic process [33]. Therefore, we speculated that STC-1 might regulate the tumor development through enhancing tumor angiogenesis. This hypothesis was confirmed by *in vivo *and *in vitro *angiogenesis experiments.

Based on these results, we proposed the below model for STC-1-mediated oncogenesis. STC-1 has no direct effect on the proliferation of cancer cells. It promotes tumor angiogenesis which in turn changes tumor microenvironments. The altered microenvironment induces the sprouting of new blood vessels from the established vasculature, resulting in a tumor vascular system. This tumor vascular system enables tumor cells to obtain enough oxygen and nutrients for survival and proliferation.

It is well recognized that VEGF is regulated by many pathways such as phosphorylated PKCβII, phosphorylated P38, and phosphorylated ERK1/2 [19,21,25]. We found STC-1 could activate PKCβIIand ERK1/2 proteins rather than P38. Blocking PKCβII or ERK1/2 reversed the expression of VEGF induced by STC-1, indicating that STC-1 regulates VEGF expression through PKCβII or ERK1/2 pathways. Moreover, we found that a combination of PKC and ERK1/2 inhibitors has the similar effect as PKCβII inhibitor itself (Figure4E). This may indicate that the ERK signaling pathway is a potential PKCβII target, which is agreement with other studies [34]. However, previous studies have proved that VEGF could regulate STC-1 expression. This may indicate that there may be a positive feedback regulation between STC-1 and VEGF.

## Conclusions

Our study showed that STC-1 promotes the expression of VEGF depended on the activation of PKCβII and ERK1/2 pathways. VEGF subsequently enhances tumor angiogenesis which in turn promotes the gastric tumor growth.

## Materials and methods

### Material

PD98059 (selective inhibitor of ERK signaling pathway) and CGP53353 (selective inhibitor of PKCβII signaling pathway) were obtained from TOCRIS Bioscience Company (Bristol, UK) and Beyotime Institute of Biotechnology (Haimen, China), respectively. Stanniocalcin-1 monoclonal human antibody was obtained from R&D Company. VEGF Rabbit Monoclonal Antibody (Bioactive), which can block ligand-receptor interaction, was obtained from Epitomics Company. Cell Apoptosis kit was obtained from MBL International Corporation (Watertown, MA). The recombinant human stanniocalcin-1 protein was obtained from PROSPEC (Protein Specialists) Company. Celltrace™ CFSE cell Proliferation kit (C34554) was obtained from Invitrogen Company.

### Cells and cell culture

Human gastric adenocarcinoma cell line BGC823 and Human umbilical vein endothelial cells (HUVECs) were obtained from Shanghai Institute of Cell Biology (Shanghai, China). BGC823 cells were cultured in RPMI1640 medium (Gibco, USA) supplemented with 10% fetal bovine serum (FBS) (Gibco, USA), 10 mg/ml streptomycin and 10,000 units/ml penicillin. G418 sulate (Merck, German) was additionally added in BGC/STC (STC-1 high expression) and BGC/shSTC cells (STC-1 low expression. HUVECs were grown in RPMI1640 medium supplemented with 10% FBS. Cells were incubated in a humidified atmosphere of 5% CO_2 _at 37°C.

### Plasmids construction and transfection

STC-1 cDNA, acquired from human gastric carcinoma tissues, was purified, digested, and ligated to pcDNA3.1 vector. Three siRNAs fragments targeted STC-1 were designed by online software http://rnaidesigner.invitrogen.com/rnaiexpress/. The most effective siRNA fragment was converted to shRNA and then was inserted into pSiencer4.1. PcDNA3.1/STC-1 and pSilencer4.1/STC-1-shRNA plasmids were constructed and transfected into BGC823 cells with Lipofectamine 2000 reagent according to the manufacturer's instructions.

### Tumor culture supernatants collection

BGC cell, BGC/CON cell, BGC/STC cell, BGC/shCON cell, and BGC/shSTC cell were seeded at 5 × 10^5 ^cells/well in triplicate on 6 well plates with 10% FBS-1640 medium, refreshed medium with serum-free 1640 medium. After 24 h, the culture supernatants were collected, centrifuged at 4°C, 4000 rcf for 10 min, and stored at -70°C for subsequent use. Tumor supernatants were labeled as BGC supernatant, BGC/CON supernatant, BGC/STC supernatant, BGC/shCON supernatant, and BGC/ shSTC supernatant.

### CFSE staining and proliferation experiments

Cells were labeled with 5-(and -6) carboxyfluorescein diacetate succinimidyl ester (CFSE; Molecular Probes, Invitrogen, USA) according to the manufacturer's protocol. A 5 mM stock solution of CFSE was prepared by dissolving in DMSO and stored at -20°C. Before labeling, cells were washed and re-suspended in PBS containing 0.1% BSA (PBS/BSA). CFSE was then added into the cell suspensions at a final concentration of 5 μM, and incubated for 15 min at 37°C. The cells were subsequently washed with complete RPMI 1640 medium and re-suspended in complete RPMI 1640 medium for culture. After incubation for days 3, the cells were harvested for the division analysis of CFSE-labeled cells by FACS.

### Xenografts experiments

Female BALB/c nude mice (5-6 weeks old) were obtained from Military Medical Sciences Laboratory Animal Research Center (Beijing, China). 10^6^cell/100 μL PBS were injected subcutaneously into the flank of female nude mice (n = 6). Tumor volumes were measured once every two days when tumors can be observed and calculated by the formula: Volume = (width)^2^× length/2.

### Immunohistochemistry analysis

Tumor tissues were harvested, fixed in 10% buffered formalin, dehydrated, bisected, mounted in paraffin, and sectioned for immunohistochemistry (IHC). Hydrated sections were stained using Hematoxylin/Eosin. IHC was carried out with antibodies specific for PCNA (Proliferating Cell Nuclear Antigen) using rabbit anti-mouse PCNA (1:1600, Dako Cytomation, Denmark) or Monoclonal Anti-human Stanniocalcin-1 antibody (R&D Systems, Inc.). The quantitation of PCNA density was normalized to the Integrated Optical Density (IOD) of PCNA via Image Pro Plus software. All histology was carried out on multiple sections from individual mice and three independent in vivo experiments.

### HUVEC migration assay

The assay was performed using cell culture inserts (8 μm pore size) (Millipore Cell, US). 2 × 10^4 ^HUVEC cells/well were seeded onto inserts with serum-free RPMI 1640 medium in triplicate. Then they were put into a 24-well culture plate containing 500 μl tumor supernatants. 12 h later, the inserts were removed and washed with PBS, fixed, stained, rinsed with water, and photographed in 3 random fields (400×, or 200×) per insert under upright microscope.

### HUVEC tube formation assay

6 × 10^4 ^HUVEC cells were seeded in triplicate on Matrigel coated 24-well plates in 500 μl RPMI 1640 with 10% FBS, cultured at 37°C. Cell culture medium was then replaced by 500 μl tumor supernatants. After 12 h, tube formations were observed under upright microscope. Tube-like structures were defined as endothelial cord formations that were connected at both ends and the mean tube length in five random fields per well was quantified.

### In vivo angiogenesis assay

Matrigel were carefully mixed with tumor cells and 64U/ml heparin. Matrigel mixtures (0.1 ml, 5 × 10^5 ^cells) were injected subcutaneously into the armpit region of 6-week-old female BALB/c nude mice. At day 14, Matrigel plugs were removed and sectioned for Hematoxylin/Eosin, the vascularity was calculated in five random fields per section by OlyVIA software and Image-Pro Plus software.

### Western Blot

Western blot analysis was performed using antibodies against anti-PKCβII and anti-PKCβII Phospho rabbit monoclonal antibody (Epitomics, CA, USA) diluted at 1: 1000, the monoclonal antibody anti- ERK1/2 and anti-ERK1/2 Phospho, anti- P38 and anti-P38 Phospho (Cell Signaling Technology, USA) at 1: 1000, and the anti-βtubulin rat monoclonal antibody (Beyotime, China) at 1:1000.

### VEGF Assay

VEGF content in tumor culture supernatants was quantified by an enzyme-linked immunosorbent assay (ELISA) kits (DAKEWE Company, China) according to the manufacturer's instructions. All assays were duplicated.

### Statistical Analysis

All results are presented as means ± S.E.M of at least three independent experiments, unless otherwise indicated. Student's t test was employed to assess differences between two groups. A value of p < 0.05 was considered to be statistically significant.

## Abbreviations

ERK1/2: extracellular signal-regulated protein kinase ½; HUVEC: Human umbilical vein endothelial cell; PCNA: Proliferating Cell Nuclear Antigen; PKCβII: intracellular protein kinaseβII; STC-1: Stanniocalcin-1; VEGF: Vascular endothelial growth factor.

## Competing interests

The authors declare that they have no competing interests.

## Authors' contributions

YYH LFH YHH conceived and designed the experiments. LFH QYG GFZ YHH performed the experiments. LFH participated in the design of the study and performed the statistical analysis. TTW LFH YYH Wrote the paper. All authors read and approved the final manuscript.

## Supplementary Material

Additional File 1 **Construction of plasmids and verification of transfected BGC cells**. (A) Cells were transiently transfected with STC-1 siRNA#1, STC-1 siRNA#2, STC-1 siRNA#3 for 24 h. Whole-cell lysates were analyzed for the levels of STC-1 by RT-PCR. (B) the expression of STC-1 in BGC823 after transfection was confirmed by RT-PCR analysis. (C) Cellular phenotypes after stable transfection. (D) Proliferation of all BGC and transfected BGC cells (5 × 10^4 ^cells/well) were determined by FACS, CFSE positive cells were gated and CFSE fluorescence intensity was showed in histograms. (E) Cell apoptosis of all BGC and transfected BGC cells. Apoptotic cells were stained using the Annexin V-FITC Apoptosis Detection Kit following the manufacturer's instruction. Click here for file
