# Supplementary material for: Stanniocalcin-1 promotes tumor angiogenesis through up-regulation of VEGF in gastric cancer cells
Source: J Biomed Sci. 2011 Jun 14;18(1):39. doi: 10.1186/1423-0127-18-39 (PMC3142497; doi:10.1186/1423-0127-18-39)
Supplement: Additional File 1 — Construction of plasmids and verification of transfected BGC cells. (A) Cells were transiently transfected with STC-1 siRNA#1, STC-1 siRNA#2, STC-1 siRNA#3 for 24 h. Whole-cell lysates were analyzed for the levels of STC-1 by RT-PCR. (B) the expression of STC-1 in BGC823 after transfection was confirmed by RT-PCR analysis. (C) Cellular phenotypes after stable transfection. (D) Proliferation of all BGC and transfected BGC cells (5 × 10^4^ cells/well) were determined by FACS, CFSE positive cells were gated and CFSE fluorescence intensity was showed in histograms. (E) Cell apoptosis of all BGC and transfected BGC cells. Apoptotic cells were stained using the Annexin V-FITC Apoptosis Detection Kit following the manufacturer's instruction. [file 1423-0127-18-39-S1.DOC]

**
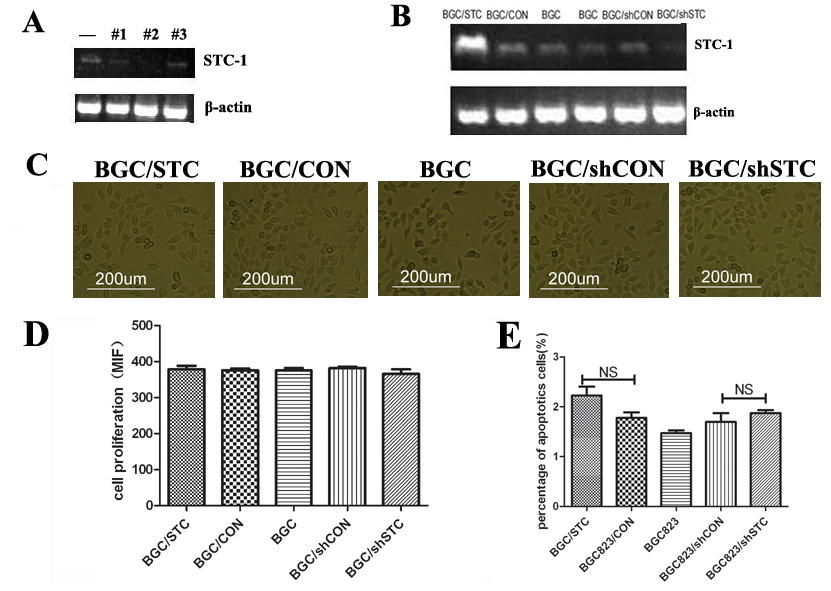
**

**Supplemental Figure** Construction of plasmids and verification of transfected BGC cells. (A) Cells were transiently transfected with STC-1 siRNA#1, STC-1 siRNA#2, STC-1 siRNA#3 for 24 h. Whole-cell lysates were analyzed for the levels of STC-1 by RT-PCR. (B) the expression of STC-1 in BGC823 after transfection was confirmed by RT-PCR analysis. (C) Cellular phenotypes after stable transfection. (D) Proliferation of all BGC and transfected BGC cells (5×10^4^ cells/well) were determined by FACS, CFSE positive cells were gated and CFSE fluorescence intensity was showed in histograms.. (E) Cell apoptosis of all BGC and transfected BGC cells. Apoptotic cells were stained using the Annexin V-FITC Apoptosis Detection Kit following the manufacturer’s instruction.
